# Surgical Repair of a Giant Sinus of Valsalva Aneurysm with an Anomalous Left Circumflex Artery in a Patient with Bicuspid Aortic Valve

**DOI:** 10.1155/2021/8825110

**Published:** 2021-01-14

**Authors:** Kristin Stawiarski, Asiya Mamhut, Elenita Kanin, Stuart Zarich

**Affiliations:** Yale New Haven Health Heart and Vascular Center, Bridgeport Hospital, Bridgeport, CT, USA

## Abstract

Congenital cardiac abnormalities are not always found in isolation. We describe a case of a giant right coronary sinus of Valsalva aneurysm with anomalous left circumflex artery in a 46-year-old male with bicuspid aortic valve and prior ventricular septal defect repair.

## 1. Introduction

Sinus of Valsalva aneurysms (SOVAs) account for only 0.09% of congenital or acquired cardiac defects [1]. However, when present, they may be associated with other cardiac anomalies [1].

## 2. Clinical Summary

A 46-year-old male with ventricular septal defect repair at age 3, bicuspid aortic valve, diabetes mellitus, hypertension, and previous tobacco use presented to clinic for routine follow-up. He was first diagnosed with an ascending aortic aneurysm 13 years prior and remained asymptomatic with a stable echocardiographic measurement of 5 cm. There was only mild aortic and mitral regurgitation with no residual shunt post-VSD repair. Electrocardiogram revealed normal sinus rhythm with incomplete right bundle branch block, right axis deviation, and poor R wave progression.

**Figure 1:**
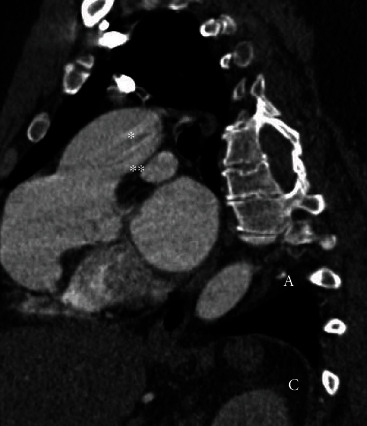
Transverse computed tomography angiography view of the aneurysm with a 2-dimensional diameter of 4.7 cm at the level of sinotubular junction.

**Figure 2:**
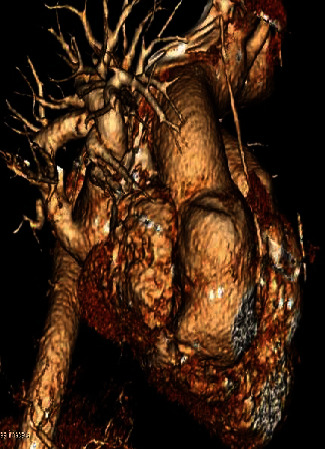
Computed tomography angiography of the sinus of Valsalva aneurysm, sagittal view. The right and noncoronary cusps are fused, separated by a raphe.

**Figure 3:**
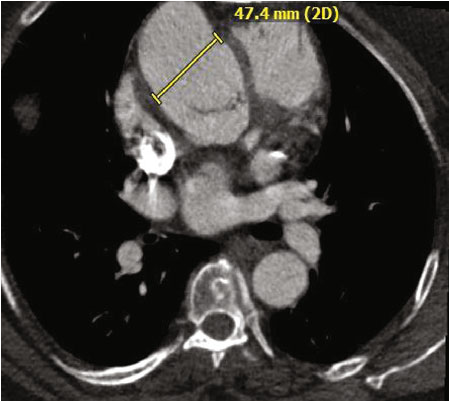
3-dimensional reconstructed view of the sinus of Valsalva aneurysm and tortuous aortic arch.

Computed tomography angiography (CTA) was obtained for further assessment. A SOVA was identified measuring 3.5 × 4.7 × 6.9 cm at the level of the sinotubular junction (Figures 1 and 2). Immediately to the aneurysm, the ascending aorta measured 4.5 × 3.7 cm. The aortic arch was markedly tortuous (Figure 3). Suspicion was raised for possible anomalous coronary artery origin; however, gated acquisition was not performed.

Surgery was planned, and preoperative angiography was completed. Aortography demonstrated a giant SOVA with a fused right and noncoronary cusp and a small appearing left coronary sinus (Video 1). The coronary system was right dominant with the right coronary and left main arteries originating from their respective cusps. The left main artery gave rise to a left anterior descending and a left circumflex artery. An ectopic duplicate left circumflex artery was also seen coming off the right coronary sinus (Video 2). All vessels were free from significant disease.

The patient underwent redo sternotomy with SOVA and ascending aorta resection. A 27 mm Hemashield valved conduit, with St. Jude mechanical valve, was sewn in place along with correct reimplantation of coronaries including the anomalous left circumflex artery.

## 3. Discussion

SOVA can manifest at any age usually with a male predisposition. These aneurysms can coexist with other congenital abnormalities such as VSD (30-60%), bicuspid aortic valves (10%), and coronary anomalies [1]. The anomalous origin of the circumflex artery from the right coronary sinus was first described in 1933 and has since had a reported incidence of 0.35% [2]. This variant carries a low risk of sudden cardiac death albeit the risk of coronary artery disease remains debatable [2].

Coronary angiography is considered the gold standard for diagnosing a SOVA [1]. However, up to 90% of cases published in the past 10 years have been diagnosed via echocardiography [1]. This diagnostic method proves to be suboptimal in evaluating rare concomitant coronary anomalies as seen with our patient [3]. The American College of Cardiology/American Heart Association (ACC/AHA) 2008 recommendations for the management of adult congenital heart disease recommend the use of CTA or magnetic resonance imaging (MRI) for initial congenital coronary anomaly screening in patients who *survived unexplained cardiac death*, *life-threatening arrhythmia*, *coronary ischemic symptoms*, *or left ventricular dysfunction* (*class I*, *level of evidence* (*LOE*) *B*) [4]. However, the more recent ACC/AHA 2018 guidelines have removed this recommendation as the data supporting a surgical approach for secondary prevention of sudden cardiac death are limited [5]. These guidelines offer coronary angiography, CTA or, MRI as the choice image modalities for the evaluation of coronary anomalies (class I, LOE C-limited) [5].

It is generally accepted to apply aortic root aneurysm repair guidelines to SOVA repair as was done in our patient. Surgical repair of the aneurysm is usually considered if the aortic diameter is ≥5.5 cm in asymptomatic patients with a bicuspid aortic valve. Exceptional consideration is given to patients who have diameter of ≥5.0 cm with either additional risk factors for dissection or an expansion rate of ≥0.5 cm per year. Likewise, aneurysm repair is reasonable in patients undergoing aortic valve replacement due to severe bicuspid valve stenosis/regurgitation [6].

A thorough evaluation of the coronary anatomy is vital prior to any cardiac surgery. Failure to recognize coronary anomalies can result in intraoperative complications such as accidental vessel laceration or compression. However, primary surgery for anomalous coronary arteries is usually reserved for cases of ischemia (class I, LOE B nonrandomized) or ventricular arrthymias (class IIA, LOE C—expert opinion), both of which are lacking in our patient [5]. Surgery would also be reasonable if the left coronary artery arises from the right coronary sinus in the absence of symptoms (class IIA, LOE C—limited data) [5]. In our case, decision was made to relocate the anomalous artery to its usual course to reduce the potential risk of coronary artery disease from angular vessel insertion.

For patients with an identified congenital cardiac abnormality, it is prudent to comprehensively evaluate the entire cardiac anatomy for other clustered anomalies as seen in this case. The risk of ischemic events and sudden cardiac death from ectopic coronary arteries remains unclear but can pose a surgical risk for patients undergoing other cardiac surgery.

## Supplementary Materials

Supplementary 1 Video 1 Aortography showing a giant sinus of Valsalva aneurysm. The right and noncoronary cusps are fused, separated by a raphe.

Supplementary 2 Video 2 Coronary angiogram of the right coronary artery and anomalous left circumflex artery arising from the right coronary cusp.
